# Diurnal Flight Activity of House Flies (*Musca domestica*) is Influenced by Sex, Time of Day, and Environmental Conditions

**DOI:** 10.3390/insects11060391

**Published:** 2020-06-23

**Authors:** Levi K. Zahn, Alec C. Gerry

**Affiliations:** Department of Entomology, University of California Riverside, Riverside, CA 92521, USA; alecg@ucr.edu

**Keywords:** temperature, light intensity, wind speed, trap, dairy, California

## Abstract

House flies (*Musca domestica* L.) are common synanthropic pests associated with confined animal operations, including dairy farms. House flies can cause substantial nuisance and may transmit human and animal pathogens. Surprisingly little is known about the daily flight activity of house flies. This study examined diurnal house fly flight activity on two southern California dairies using clear sticky traps to capture flies over hourly intervals. Flight activity for both males and females combined started near dawn and generally increased to a single broad activity peak during mid to late morning. Male flight activity peaked earlier than female flight activity and this separation in peak activity widened as mean daytime temperature increased. Flight activity for both sexes increased rapidly during early morning in response to the combined effects of increasing light intensity and temperature, with decreasing flight activity late in the day as temperature decreased. During midday, flight activity was slightly negatively associated with light intensity and temperature. Collection period (time of day) was a useful predictor of house fly activity on southern California dairies and the diurnal pattern of flight activity should be considered when developing house fly monitoring and control programs.

## 1. Introduction

House flies can be a significant source of nuisance, on occasion prompting legal action, as flies disperse from concentrated development sites into surrounding neighborhoods [1,2,3]. House flies are also capable of mechanically transmitting pathogens that impact both human and animal health [4,5,6,7]. The daily pattern of house fly flight activity (including dispersal flight) likely impacts both their potential to cause nuisance and to spread pathogens.

House flies are diurnally active, moving to overnight resting sites late in the day where they remain inactive at night [8,9]. However, published reports describing the daily pattern of house fly flight activity are conflicting, with daily flight activity reported as either unimodal (single peak in activity) or bimodal (two peaks in activity). In India, flight activity was unimodal during June with peak flight in early to mid-afternoon [10]. In Japan, flight activity was unimodal during spring with a peak in late morning but was bimodal during summer with activity peaks in both the morning and afternoon [11]. Flight activity was bimodal in Kansas during summer [12] and in Sudan from late summer through winter [13]. West [8] suggests that flight activity in house flies may shift seasonally, perhaps in response to changing daytime temperature, with a single peak in flight activity during cool months and a bimodal pattern in flight activity during hot months, but this hypothesis remains untested. Differences in the timing of peak flight activity are also reported between fly sexes, with flight activity for males often occurring earlier in the day than for females [13].

Taylor [14] postulated that the onset flight activity of diurnal insects is a function of both temperature and light intensity, with flight activity occurring if temperatures exceed a species-specific minimum threshold and increasing in association with increasing light intensity near dawn. However, the specific relationship of house fly flight activity with temperature or light intensity is not well understood and is likely confounded by other environmental variables [15]. Inside a dairy barn, house fly flight activity was positively correlated with temperatures up to 42 °C, the maximum temperature observed [12]. Flight activity was negatively correlated with humidity in a laboratory study [16]. The influence of wind speed on house fly flight activity has not been reported, perhaps because wind speed estimation is challenging due to extreme spatial and temporal variability. However, it has been proposed that most flying insects will reduce flight activity when wind speeds exceed the maximum flight speed for the species [17,18].

The variability in house fly flight activity within and between days may impact the accuracy of house fly abundance estimates using traps or instantaneous visual counts [19]. This is particularly true when monitoring methods are deployed for only a few hours each day or when the time that monitoring is performed varies among days. Studies to better understand diurnal house fly flight activity over a range of environmental conditions are needed to improve house fly surveillance by targeting the period of greatest flight activity each day. Determining the peak flight activity period can also improve house fly management programs by suggesting target periods to maximize insecticide contact with active adult flies when applying non-persistent insecticides as a mist, fog, or ultra-low volume (ULV) spray [20]. 

The current study determines the relationship of house fly flight activity on two commercial dairies in southern California with time of day and environmental conditions including light intensity, temperature, humidity, and wind speed during the summer when house fly abundance is highest and management of house flies is of greatest concern.

## 2. Materials and Methods

**Figure 1:**
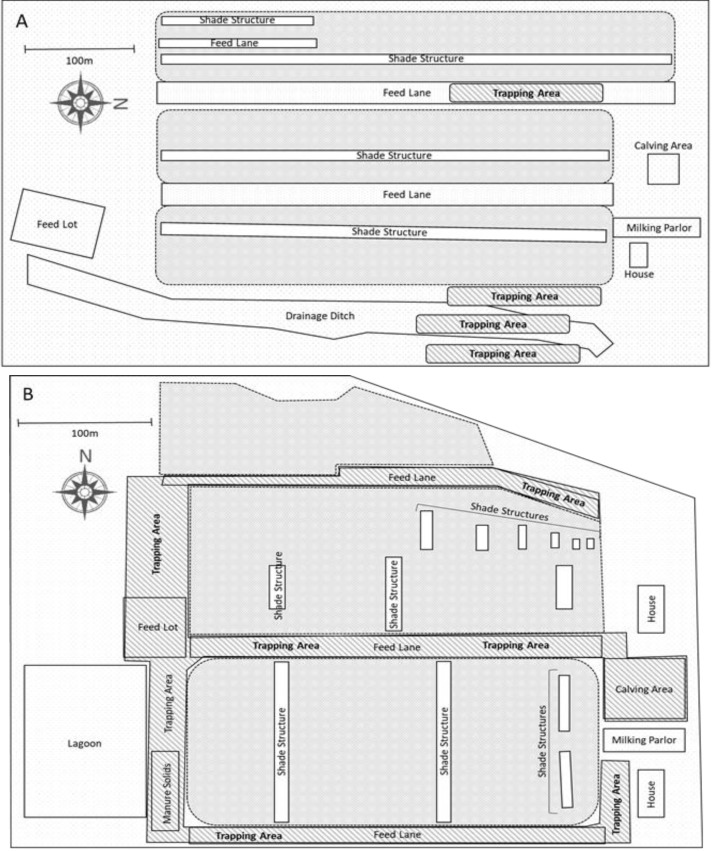
Diagrammatic sketches of the dairies and sites sampled. Cattle are held in separate pens collectively indicated by areas with solid grey shading. (**A**) BS Dairy: Three traps were placed into each of 4 trapping areas (dark grey diagonal striped area) ranging from just west of the dairy center to the eastern edge of the dairy (12 traps per day). (**B**) OS dairy: Twelve traps were placed randomly each day within the trapping area (dark grey diagonal striped area), at locations where facility workers and cattle would not interfere with them.

Diurnal flight activity of adult house flies during the seasonal period of peak activity was examined on two large active dairy facilities (BS dairy and OS dairy; Figure 1) near the southern California town of San Jacinto. The dairies are approximately 3.8 km apart and are located near several other dairies of similar size. Cows at both dairies are housed in separate dry lot pens by cow age and freshening date (time since start of lactation). Pens are connected via alleys to a single milking parlor where cows are milked twice per day. Cattle movement on these dairies is therefore nearly continuous as cows in each pen are sequentially moved to the milking parlor and then return to their pen. At the BS dairy, trapping was conducted over four days in July of 2014, with one additional day in July 2017 for confirmation of the earlier results. At the OS Dairy, trapping was conducted over seven independent days from July–November 2017. 

**Figure 2:**
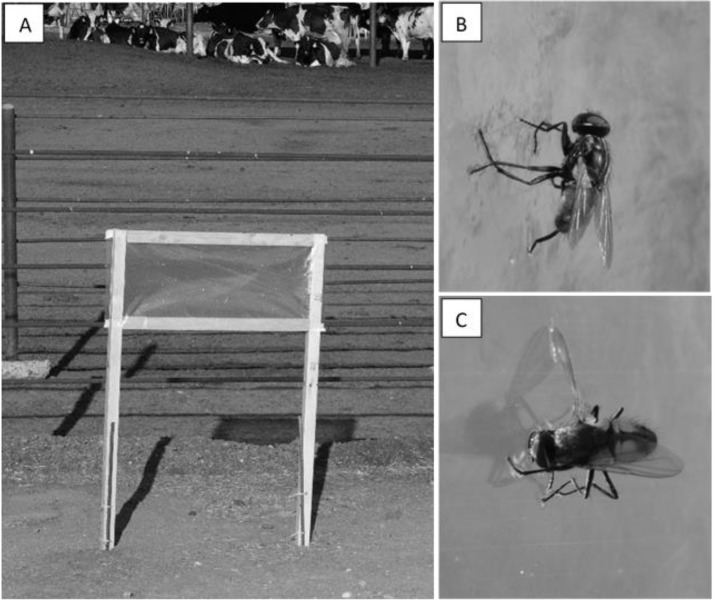
**(A**) sticky panel trap used to capture flying house flies; (**B**) house fly stuck to adhesive surface of sticky trap; (**C**) house fly that appears to have flown directly into the adhesive surface of the sticky trap.

Flight activity was recorded hourly starting from 30 min before civil dawn (ranging from 05:50–07:07 over the course of the study) through civil dusk (19:00–21:00) using 12 sticky panel traps consisting of a wooden frame (30.5 cm tall × 70 cm wide) with a clear plastic sticky sheet (Olson Products, Medina, OH, USA, Part# B005BCST8M) attached to both the front and back of the frame (Figure 2A). The top of each trap was positioned 1 m above ground and there was a minimum of 10 m separation among traps. House flies were observed to both land on (Figure 2B) and directly fly into these clear sticky sheets (Figure 2C), an indication that flies did not always perceive the clear sticky sheets as a barrier. At the BS dairy, traps were grouped into four trapping areas which were selected to avoid impacting normal dairy operations and to sample a variety of areas ranging from west of the dairy center to the eastern edge of the dairy. All traps were positioned with the sticky faces oriented east and west. Trap locations remained in the same positions over all trapping days at the BS dairy. At the OS dairy, trap placement and trap face orientation by cardinal direction was randomly selected each study day. The layout of this dairy allowed for more flexibility in trap placement than at the BS dairy.

At the end of each one-hour collection period, flies captured on the sticky traps were identified to species, sexed, counted, and removed from every trap. Thus, each hourly count provided a measure of house fly flight activity by sex during the preceding 1 h collection period. There were 13–16 collection periods per study date, depending upon the length of time from dawn to dusk on each date.

Wind speed (m/s), temperature (°C), and relative humidity (%) were averaged over each hourly interval using a Kestrel^©^ portable weather station (Kestrel Instruments, Boothwyn, PA, USA, sku# 0855) and the average was recorded at the end of each collection period. Light intensity (watts/m^2^) was collected using the California Irrigation Management Information System (CIMIS, https://cimis.water.ca.gov/) network of weather stations (#240 Perris-Menifee). Since light intensity was reported hourly, it did not always evenly match with each collection period on each date but was matched based on the hourly interval that encompassed the most of each collection period.

### 2.1. Data Analysis

For each dairy, the proportion of male and female flies captured across all traps and dates was examined using a chi-square goodness-of-fit test for equal distribution by fly sex. Dairies were analyzed separately since trap placement and collection year were different. These and all subsequent analyses were performed using R Statistical Software (R-Core Team, Vienna, Austria, version 3.5.1) [21].

#### 2.1.1. Flight Activity by Time of Day

Differences in house fly flight activity by time of day (collection period) for each dairy were evaluated using a linear mixed effect model (LMM) using the ‘nlme’ package [22] with the number of house flies (sexes combined) within each collection period for each sampling date as the response variable. The pooled number of house flies was square-root transformed before analysis to meet test assumptions. The collection date was included as a random effect since each dairy was sampled on multiple days. Due to autocorrelation of the residuals, the correlation structure of the random effect was modified to include a lag 1 auto-regressive (AR1) term [22,23,24]. The main effect of collection period was evaluated using ANOVA (type II) tests using Satterthwaite’s approximation of the denominator degrees of freedom on restricted maximum likelihood models [25].

The model assumptions of linearity and homoscedasticity of the residuals were visually checked using quantile–quantile plots (Q–Q plots) and residual versus fitted plots, respectively [23]. The presence of autocorrelation among the residuals was visually assessed using the ‘stats’ package [21]. Model overdispersion was checked using both the residual versus fitted plot and using the method outlined in [26] where the sum of the squared Pearson residuals should be χ^2^ distributed. For LMMs the conditional R-squared (R^2^c) was used as a metric of model quality and was calculated for each mixed effect model using the R package ‘piecewiseSEM’ [27]. The conditional R-squared is an estimate of the total variation explained by both the fixed and random effects in the model.

Variation in house fly sex ratio among collection periods for each dairy was examined using a generalized linear mixed effect model (GLMM) using the R package ‘lme4′ since the response variable was binomial [28]. Collection period was included as a fixed effect and date of collection as a random effect. A binomial error distribution with a logit link function was specified. Wald χ^2^ tests were used to assess the significance of the fixed effect. An all pairwise comparison of house fly sex ratios from each collection period was performed using the R package [29], using a Tukey’s correction of the *p*-value (α = 0.05) for multiple comparisons. Model assumptions were checked using the methods described above.

An additional analysis of covariance was performed to determine whether the sex-specific time of peak flight activity was predicted by environmental factors. On each sampling date the time of peak flight activity for each sex was considered to be the collection period with the greatest total capture of flies of that sex. On one date, the same number of female flies was captured during two non-adjacent collection periods and the collection period with the greatest number of female flies in the two adjacent periods was determined to be the time of peak female flight activity. The time of peak flight activity was regressed against the average daytime values for environmental variable (temperature, relative humidity, wind speed, and light intensity) using a multiple linear regression [21]. Collection date and house fly sex were also included as main effects. The two-way interactions of house fly sex with each environmental variable and with date of collection were also included in the initial full model. Data from both dairies were combined for this analysis. The full model, including all fixed effects and two-way interaction terms, was reduced using likelihood ratio tests until a final minimally adequate model was found. Model assumptions of linearity and homoscedasticity of the residuals was visually checked using quantile–quantile plots (Q–Q plots) and residual versus fitted plots, respectively [23].

#### 2.1.2. Effect of Environmental Variables on House Fly Flight Activity

To evaluate the effect of individual environmental variables on flight activity, collection periods on each sampling date were separated into individual time of day categories: dawn (first two collection periods), dusk (last two collection periods), or midday (all remaining collection periods). Groupings were based upon similar overall flight activity patterns observed during these periods. For each time of day category, the number of house flies captured was pooled, by sex, over all traps within a collection period and sampling date to give a single fly capture value for each collection period from each date. Dairies were combined for this analysis. Linear mixed effect models, using the R package ‘nlme,’ [22] were created to assess the impact environmental variables had on house fly flight activity. House fly sex, temperature, light intensity, relative humidity, wind speed, and their two-way interactions with house fly sex were included as fixed effects, while date of collection was included as a random effect. The number of house flies, by sex, was cube-root transformed to improve normality before analysis. Models were reduced using likelihood ratio tests. The significance of individual model coefficients was assessed using Wald χ^2^ tests [25].

## 3. Results

A total of 9233 house flies were captured during this study, with 3572 house flies captured at the BS dairy and 5661 house flies captured at the OS dairy. The sex ratio of captured flies was significantly skewed towards male flies at both dairies, with males comprising 55.9% of total flies captured at the BS Dairy and 59.5% of flies captured at the OS Dairy (χ^2^ = 49.86; df = 1; *p* < 0.001 and χ^2^ = 204.9; df = 1; *p* < 0.001, respectively).

### 3.1. Flight Activity by Time of Day

**Figure 3:**
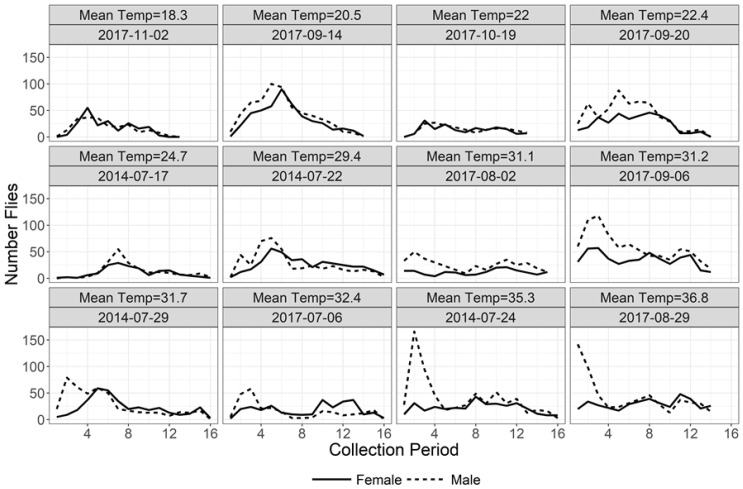
Total female (solid) and male (dotted) house flies collected during each collection period on each date. Panels are arranged from lowest (top left) to highest (bottom right) mean daytime temperature.

**Figure 4:**
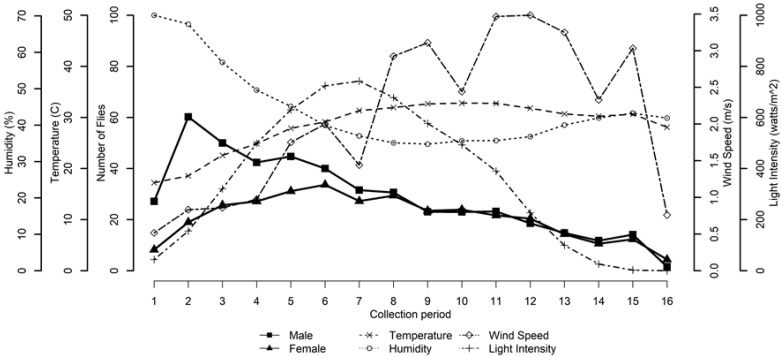
Number of male (square) and female (triangle) house flies collected in relation to environmental variables for each collection period when averaged over all collection dates. Figure 4 illustrates how fly activity and environmental variables generally change over the course of a day.

While there was variation in the magnitude and timing of flight activity across collection dates, flight activity for both males and females was generally unimodal with males exhibiting an early morning activity peak while females exhibited a less pronounced activity peak later in the day particularly as mean daytime temperature increased (Figure 3). This unimodal pattern of flight activity for each fly sex is more clearly observed when flight activity was averaged across all collection dates (Figure 4). Observed environmental data indicate that light intensity follows a predictable unimodal pattern with a midday peak, temperature and humidity move in opposition with an inflection point in mid-afternoon, and wind speed is lowest at dawn and increases to irregular peaks in late afternoon.

**Figure 5:**
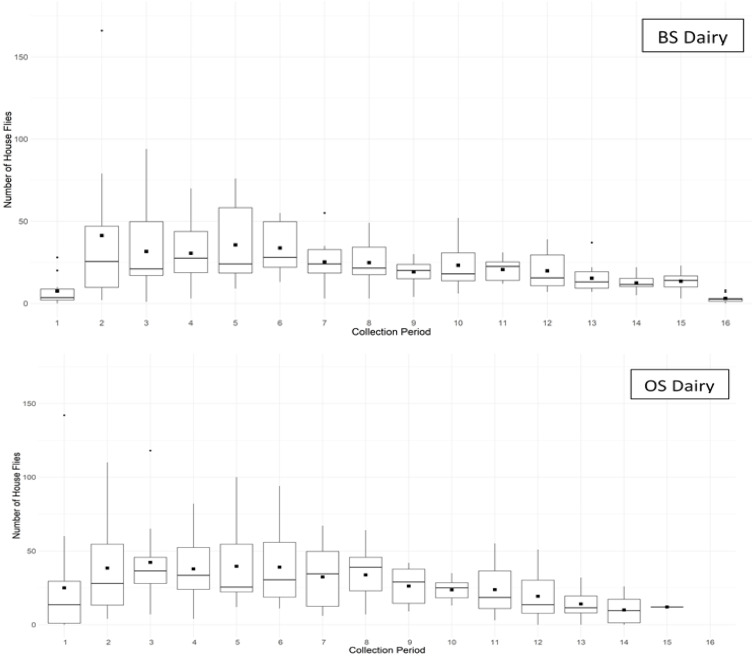
Box plots showing house fly activity for combined sexes by collection period across all sampling dates at the BS dairy and OS dairy. Collection periods were hourly intervals starting from 30 min before civil dawn through civil dusk. Due to shortened day length on dates when the OS dairy was sampled, this dairy lacked period 16 collections. Black squares show the average number of house flies collected.

**Figure 6:**
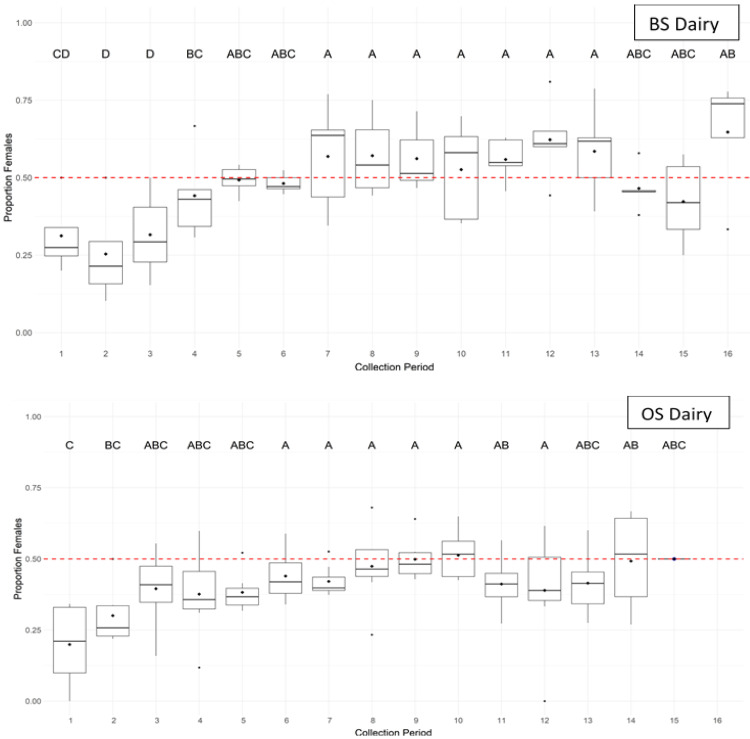
Proportion of house flies captured on sticky traps that were female during each collection period at the BS Dairy and OS Dairy. Black diamonds indicate raw mean proportions. Sex ratio was not significantly different after Tukey’s correction of the *p*-value (α = 0.05) for collection periods with the same letter.

Considering both male and female flies together, flight activity was significantly related to time of day (collection period) at both the BS dairy (F_15,140_ = 6.70; *p* < 0.001; R^2^c = 0.41) and the OS dairy (F_14,173_ = 7.28; *p* < 0.001; R^2^c = 0.49), with flight activity beginning near civil dawn, followed by a single peak in flight activity during mid to late morning (Figure 5). However, the sex ratio of captured flies varied significantly by time of day at both the BS dairy (Wald: χ^2^_15_ = 245.21; *p* < 0.001) and OS dairy (Wald: χ^2^_14_ = 119.91; *p* < 0.001). Proportionally fewer female flies were captured during the first three hourly collection periods (up to 2.5 h after dawn) at the BS dairy (<31.5% female, *p* < 0.02) and the first two hourly collection periods (up to 1.5 h after dawn) at the OS dairy (<30.0% female, *p* < 0.05), with female flies comprising closer to 50% of captured flies during all later collection periods at both dairies (Figure 6). 

**Figure 7:**
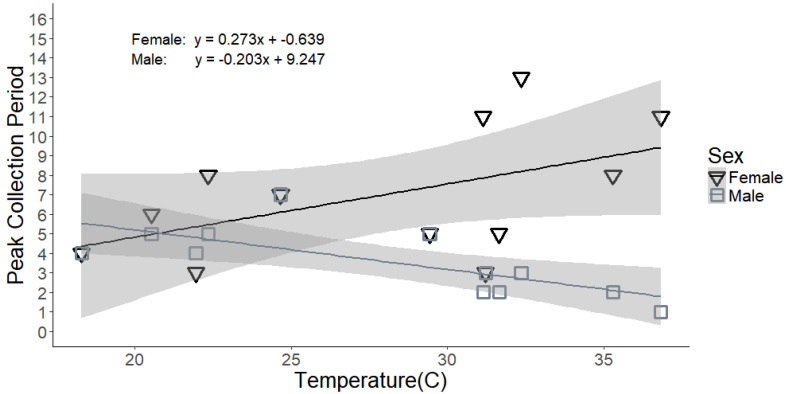
Collection period with peak house fly flight activity by mean daytime temperature for male (grey; squares) and female (black; triangles) house flies. Shaded bands around each line indicate the 95% C.I. by fly sex.

The time of peak flight activity also differed for male and female flies (F_1,20_ = 13.35; *p* = 0.002). Male peak flight activity occurred approximately 1–3 h after sunrise (mean ± SE collection period = 2.60 ± 1.10) while female peak flight activity occurred over a broader time range later in the day at approximately 3–8 h after sunrise (mean ± SE collection period = 6.01 ± 2.72) (Table 1). The greater male flight activity during early morning is particularly noticeable when flight activity is separated by sex and collection date (Figure 3). Although time of peak flight activity was not significantly related to mean daytime temperature overall (F_1,20_ = 0.20; *p* = 0.662), there was a significant interaction of house fly sex with mean daytime temperature (F_1,20_ = 8.94; *p* = 0.007). Each 1.0 °C increase in mean daytime temperature was associated with a 0.20 h earlier shift in peak flight activity for males but a 0.27 h later shift in peak flight activity for females resulting in greater separation of peak flight activity between the sexes as temperature increased (Figure 7). Male peak flight activity was particularly responsive to changes in mean daytime temperature with a much narrower confidence interval relative to female peak flight activity. The shift toward earlier male flight activity with increasing temperature is especially notable when examining fly captures for all collection dates arranged from lowest to highest mean daytime temperature (Figure 3). Light intensity, relative humidity, wind speed, and date of collection did not significantly affect the time of peak flight activity and were removed from the final model (data not shown).

### 3.2. Effect of Environmental Variables on Flight Activity

**Figure 8:**
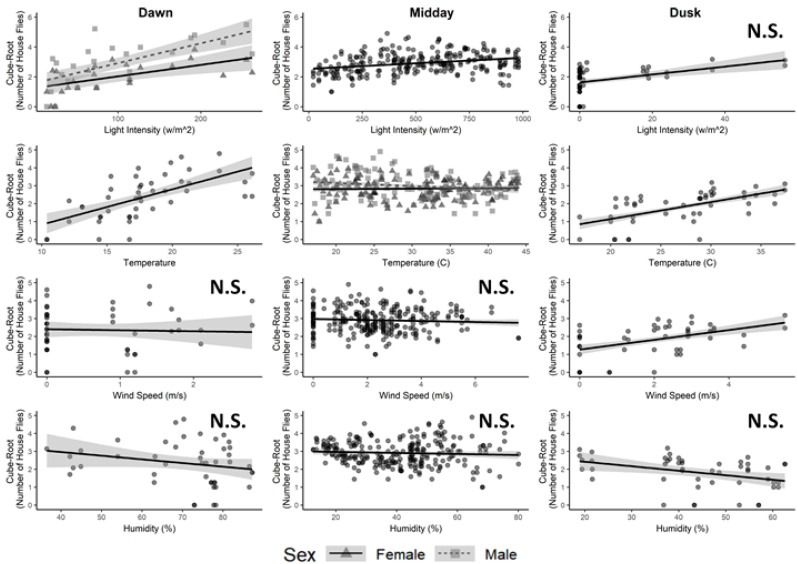
Predictors of house fly activity near Dawn, through Midday, and near Dusk. Each panel shows the relationship of house fly activity with measured environmental variables: light intensity, temperature, wind speed, and humidity. For environmental variables to which the fly sexes responded similarly, data are combined into single regression line with data points indicated as grey circles. When significant differences by sex were observed for an environmental variable, the data are separated for males (dashed lines; squares) and females (solid line; triangles). Light grey bands around each line indicate the 95% C.I. for each fitted regression line. Non-significant environmental factors are indicated by N.S.

The assignment of hourly collection periods on each collection date into one of three time of day groupings (Dawn, Midday, or Dusk) proved satisfactory to model the effect of environmental variables on house fly flight activity over the course of a day (Dawn: R^2^c = 0.88; Midday R^2^c = 0.55; and Dusk R^2^c = 0.67). Generally, male and female house flies responded similarly to changing environmental variables except for light intensity near dawn and temperature during midday (Table 2 and Figure 8). At dawn, flight activity for both males and females was positively associated with light intensity, but male flight activity increased with light intensity at a much more rapid rate. At midday, flight activity for both males and females was negatively associated with temperature, but male flight activity decreased with temperature at a greater rate.

At Dawn, flight activity for both sexes was positively associated with light intensity and temperature, while unrelated to both wind speed and humidity. During Midday, flight activity for both sexes was positively associated with light intensity but negatively associated with temperature, while unrelated to wind speed and humidity. At Dusk, flight activity for both sexes was positively associated with temperature and wind speed, while unrelated to light intensity or humidity.

Combining environmental associations across a typical day, flight activity for both sexes increased rapidly during early morning in response to the combined effects of increasing light intensity and temperature, with only modest change in flight activity related to light intensity and temperature during midday, followed by decreasing flight activity late in the day as temperature was reduced.

The significant positive association of flight activity with wind speed at dusk seems counterintuitive, but likely reflects the cessation of both wind and fly activity following dusk. The lack of association for flight activity with light intensity at dusk is also unexpected but may reflect both the low range of light intensity and the many very low light intensity measurements recorded during the last two collection periods comprising the dusk period, particularly as environmental data were recorded at the end of each collection period, with the last collection period ending after civil dusk.

## 4. Discussion

### 4.1. Diurnal Flight Activity

House fly flight activity varied in magnitude and timing of peak activity for individual collection dates, but when all dates are considered together a general pattern of flight activity is evident. In agreement with previous studies, house fly flight activity started near dawn [8,12,13] and generally increased to a single broad activity peak encompassing the flight activity of both male and female flies [10]. In the current study, this broad activity peak occurred in mid-morning through early afternoon over the range of summer and fall dates examined. Some previous studies reported house fly flight activity to be bimodal [12,13,30] or unimodal in winter and bimodal in summer [11]. West [8] suggested that house flies might adjust to a bimodal flight activity pattern during hotter summer months to avoid higher midday temperatures during summer. However, in the current study, house fly activity was generally unimodal even during the very hot summer months in southern California.

Studies reporting a bimodal house fly activity pattern [11,12,30] did not determine sex-specific flight activity. However, males and females have been reported to vary in their time of activity [13] and the timing of peak flight activity for male and female house flies varied considerably in the current study. Males dominated early morning collections, with male flight activity usually increasingly rapidly after dawn to a clearly discernable single peak within 1–3 h after dawn. In contrast, females were greatly underrepresented during early morning collections, with female flight activity increasing slowly to a broad and often indiscernible peak later in the day or on some occasions producing no clear female activity peak during the day. The sex-specific difference in time of peak flight activity particularly on the hottest days could result in the overall flight activity pattern appearing to be bimodal if flies were not separated by sex.

On days when mean daytime temperature was high (>29 °C), the male flight activity peak generally became more acute and shifted closer to dawn, while female flight activity became more irregular and with a less discernable activity peak. While Parker [13] recorded bimodal male flight activity, with well-defined activity peaks of similar magnitude in early morning and again in late afternoon, it is worth noting that his study in Sudan was performed at <50% RH while the current study was conducted at a considerable higher 45–90% RH humidity. Perhaps this difference in morning humidity impacted the morning male flight activity in these two studies. Alternatively, these differences simply reflect variation among the populations of flies used in the Parker [13] study and in the current study.

Overall, more male than female house flies were collected at both southern California dairies. Because the house fly sex ratio at emergence is nearly equal [31], the skewed sex ratio noted in the current study (56–60% males) is likely due to the greater capture of males during the early morning collection periods, especially on the two hottest days. However, the skewed sex ratio may also be due to greater female dispersal from the dairy following emergence [32] or post-copulation [33], greater activity of male flies near trap locations or at trap heights [34], greater overall flight activity of males, or males using the traps as resting or mating sites [33,35]. Following the early morning collection periods, males and females were captured in approximately similar numbers throughout the remainder of the day, with the exception that the proportion of flies that were female was slightly greater at the BS dairy relative to the OS dairy. This slight difference in sex ratio among the two dairies may be due to the trap locations selected at each dairy, as sex-specific differences in capture rates have been noted previously among different trap locations on a dairy [34].

The timing of peak flight activity for house flies of both sexes was similar at the two dairies used in this study. While both dairies are dry lot dairies of similar size and number of animals, facility differences in the relative location of cattle pens, feed storage areas, distance from pens to milking parlors, and positioning and orientation of fly traps did not alter the general pattern of house fly flight activity by sex between the two dairies. This suggests that overall house fly flight activity is not impacted by the movements or activity patterns of cattle or facility workers, but is rather driven by house fly responses to environmental factors as described below. Impacts of cattle movement or worker activities on nearby individual traps may have occurred but was not assessed in this study, and the summation of data from 12 traps per collection period was deemed sufficient to overcome any variation in a single or small number of traps that might have been caused by these activities.

### 4.2. Flight Activity Predicted by Environmental Variables

House fly activity is modulated by environmental conditions including temperature, humidity, light intensity, and wind speed [8,12,15,16,18,36], but these studies have largely been conducted under laboratory conditions [16,36] or within protected enclosures [12,15], like barns or houses, rather than in an open field environment. Relating these studies to an open field environment is difficult due to the covarying nature of environmental variables in the field (i.e., an increase in temperature is often associated with a decrease in the relative humidity). As expected, in the current study most of the environmental variables observed were correlated with one another, however only a few environmental factors had a significant impact on house fly flight activity and their covariates were therefore excluded from the final models.

The onset of house fly activity occurred near dawn, generally agreeing with previous studies [12,13]. After dawn, flight activity was strongly associated with rapidly increasing light intensity. For many insects, sunlight is the dominant cue initiating flight activity each day unless temperature is too low for flight to occur [14,37]. In the current study, the lowest temperature recorded exceeded the estimated lower temperature threshold for house fly walking and flight activity (<10 °C) [8,36].

Male and female house flies displayed differential behavioral responses when mean daytime temperature exceeded ~29 °C, above which male peak flight activity shifted to an earlier time of day and female peak flight activity shifted to later in the day or failed to show a clearly discernable peak. This difference was particularly stark on the hottest days (>35 °C) when male flight activity peaked within the first two collection periods (within 1.5 h of dawn) and then rapidly decreased thereafter. It seems that male house flies in particular are modifying their flight activity behavior to avoid exposure during the hottest parts of the day. Male mating activities (including mate location) are perhaps subject to greater heat stress than female activities, encouraging males more so than females to shift flight activity to the cooler early morning period.

While flight activity of male and females occurred over a wide range of midday temperatures, flight activity declined as temperature increased. However, a specific inflection point indicating a temperature at which flight activity began to decline could not be identified. Schou et al. [36] observed that in a controlled laboratory settings house fly walking activity increased with temperature from 10 °C through 35 °C, above which walking activity decreased, suggesting an upper threshold for house fly activity. In the current study, there was no distinct change in flight activity near this temperature.

Overall, relative humidity was not a good predictor of house fly flight activity in southern California, although it may have a stronger impact on house fly activity in tropical climates [10]. Wind speed also was not a good predictor of flight activity over most of the day, though there was a significant positive association of flight activity with wind speed near dusk. This is an unexpected result as it was anticipated that the relationship would be negative or not significant, as for many other larger bodied insect species [18]. However, the positive association of flight activity with wind speed at dusk may be spurious and simply reflect the cessation of both wind and fly activity following dusk.

## 5. Conclusions

The time of peak activity for each fly sex varied substantially across days as environmental conditions fluctuated. Therefore, any house fly monitoring method that records fly activity over a brief period of time during any single day may well miss the peak activity period, resulting in inaccuracy of relative fly abundance estimates among sampling dates. This supports recommendations to monitor house fly activity over multiple days to assess change in relative fly abundance or activity [19,38]. Although not specifically evaluated in the current study, flight activity at the study dairies might be expected to relate to the timing of house fly dispersal from these dairies. To reduce nuisance or other impacts by house flies, insecticides targeting active adult flies (e.g., ULV applications) should be applied during mid-morning hours when both sexes are most active and wind speeds are low, while insecticides targeting overnight resting sites may be most effective if applied during early morning before dawn when male activity increases.

## Figures and Tables

**Table 1:** Summary data for each study date showing the start time of the first collection period (30 min before civil dawn), the collection period and associated clock time of peak flight activity for female and male house flies, and summary statistics for environmental data collected over all collection periods on that day.

		Female		Male		Temperature (°C)			Humidity (%)			Light Intensity (w/m^2^)			Wind Speed (m/s)		
Date	Start Time	Coll. Period	Peak Time	Coll. Period	Peak Time	Min	Max	Mean ± SE	Min	Max	Mean ± SE	Min	Max	Mean ± SE	Min	Max	Mean ± SE
17-Jul-14	5:50	7	12:50	7	12:50	16.72	32.28	24.66 ± 1.42	33.40	77.90	52.3 ± 3.63	0.00	978.00	427.13 ± 97.81	1.10	7.60	3.2 ± 0.42
22-Jul-14	5:55	5	10:55	5	10:55	14.50	37.00	29.44 ± 1.81	20.40	77.10	39.4 ± 4.17	0.00	979.00	508.69 ± 91.89	0.00	4.60	2.04 ± 0.34
24-Jul-14	5:56	8	13:56	2	7:56	17.61	44.00	35.3 ± 2.21	12.60	44.90	23.81 ± 2.24	0.00	980.00	501.44 ± 92.02	0.00	5.50	2.26 ± 0.44
29-Jul-14	6:00	5	11:00	2	8:00	18.50	40.00	31.65 ± 1.63	19.20	44.10	31.08 ± 1.87	0.00	864.00	409.69 ± 78.07	0.00	4.10	1.84 ± 0.34
6-Jul-17	5:38	13	18:38	3	8:38	17.22	41.67	32.36 ± 1.94	26.10	63.00	44.18 ± 2.21	1.00	934.50	471.41 ± 87.66	0.00	3.40	1.53 ± 0.33
2-Aug-17	6:00	11	17:00	2	8:00	25.56	35.00	31.15 ± 0.78	52.80	81.70	64.39 ± 2.61	0.00	496.00	215.67 ± 46.01	0.00	3.70	1.52 ± 0.42
29-Aug-17	6:24	11	17:24	1	7:24	20.70	43.80	36.83 ± 1.99	16.00	68.30	26.54 ± 4.67	2.00	847.50	387.75 ± 75.93	0.00	6.60	2.47 ± 0.56
6-Sep-17	6:30	3	9:30	3	9:30	21.30	43.60	31.22 ± 1.51	18.30	80.20	40.69 ± 5.15	0.50	834.00	441.46 ± 82.58	0.00	5.50	2.66 ± 0.54
14-Sep-17	6:35	6	12:35	5	11:35	12.10	25.30	20.54 ± 0.99	44.50	86.50	57.47 ± 3.67	0.00	728.50	315.64 ± 61.56	0.40	5.70	2.7 ± 0.43
20-Sep-17	6:40	8	14:40	5	11:40	17.40	26.70	22.35 ± 0.78	41.30	74.80	55.98 ± 2.79	0.00	666.00	311.79 ± 66.22	0.80	4.30	2.16 ± 0.29
19-Oct-17	7:00	3	10:00	4	11:00	10.40	29.40	21.96 ± 1.52	35.70	87.20	51.01 ± 4.5	0.00	636.00	249 ± 59.94	0.00	3.10	1.17 ± 0.29
2-Nov-17	7:07	4	11:07	4	11:07	14.40	21.10	18.3 ± 0.57	43.30	78.20	54.02 ± 3.41	0.00	574.00	253.31 ± 63.5	0.00	2.90	1.4 ± 0.28

**Table 2:** Linear mixed effects models showing predictors of house fly flight activity. The response variable (number of flies) was cube-root transformed to normalize the residuals errors, values have not been back transformed. Data is separated into Dawn = first 2 collection periods, Dusk = last 2 collection periods, and Midday = all collection periods in between. Individual analyses were performed for dawn, midday, and dusk. Estimate is the size of the effect of each variable on the number of house flies captured, standard error gives the relative accuracy of this estimate, the t value is the estimate divided by its standard error, and the Pr (>|t|) gives probability of observing this result in a random set of data. The Akaike information criterion (AIC) indicates relative model quality.

Dawn	Fixed Effects	Estimate	Standard Error	t Value	Pr (>|t|)	Wald χ^2^	*p*-Value
	**Initial Model (AIC = 155.78)**						
	Intercept	−2.271	1.386	−1.639	0.113		
	Sex	0.816	1.134	0.719	0.479	42.210	0.000
	Temperature	0.176	0.051	3.428	0.002	18.220	0.000
	Light Intensity	0.005	0.002	3.018	0.006	24.590	0.000
	Relative Humidity	0.009	0.014	0.633	0.533	0.000	0.950
	Wind Speed	0.016	0.204	0.081	0.937	0.070	0.780
	Sex ^X^ Temperature	0.057	0.037	1.513	0.142	2.290	0.130
	Sex ^X^ Light Intensity	0.003	0.002	1.525	0.139	2.330	0.130
	Sex ^X^ Relative Humidity	−0.019	0.012	−1.580	0.126	2.500	0.110
	Sex ^X^ Wind Speed	0.066	0.189	0.349	0.730	0.120	0.730
	**Best Fit Model (AIC = 129.08)**						
	Intercept	−1.982	0.801	−2.476	0.014		
	Sex	0.331	0.232	1.427	0.154	40.390	0.000
	Temperature	0.199	0.045	4.394	<0.001	19.310	0.000
	Light Intensity	0.004	0.002	2.919	0.004	35.720	0.000
	Sex ^X^ Light Intensity	0.006	0.002	3.010	0.003	9.060	0.000
**Midday**							
	**Initial Model (AIC = 498.47)**						
	Intercept	2.861	0.557	5.134	<0.001		
	Sex	0.466	0.585	0.797	0.427	5.310	0.020
	Temperature	−0.026	0.011	−2.277	0.024	9.670	0.000
	Light Intensity	0.001	0.000	5.359	<0.001	49.220	0.000
	Relative Humidity	0.003	0.006	0.554	0.580	1.090	0.300
	Wind Speed	0.063	0.032	1.956	0.052	1.590	0.210
	Sex ^X^ Temperature	−0.009	0.011	−0.818	0.415	0.670	0.410
	Sex ^X^ Light Intensity	0.000	0.000	−0.295	0.769	0.090	0.770
	Sex ^X^ Relative Humidity	0.004	0.006	0.625	0.533	0.390	0.530
	Sex ^X^ Wind Speed	−0.065	0.042	−1.535	0.127	2.360	0.120
	**Best Fit Model (AIC = 451.88)**						
	Intercept	3.126	0.276	11.325	<0.001		
	Sex	0.718	0.253	2.841	0.005	5.280	0.020
	Temperature	−0.024	0.008	−2.980	0.003	23.370	0.000
	Light Intensity	0.001	0.000	6.907	<0.001	47.710	0.000
	Sex ^X^ Temperature	−0.019	0.008	−2.351	0.019	5.530	0.020
**Dusk**							
	**Initial Model (AIC = 153.29)**						
	Intercept	−0.827	1.892	−0.437	0.666		
	Sex	1.495	2.091	0.715	0.481	0.130	0.720
	Temperature	0.093	0.045	2.050	0.051	3.220	0.070
	Light Intensity	0.001	0.011	0.072	0.943	0.140	0.710
	Relative Humidity	−0.005	0.020	−0.245	0.809	0.440	0.510
	Wind Speed	0.153	0.107	1.426	0.166	10.160	0.000
	Sex ^X^ Temperature	−0.050	0.050	−1.001	0.326	1.000	0.320
	Sex ^X^ Light Intensity	0.004	0.014	0.314	0.757	0.100	0.750
	Sex ^X^ Relative Humidity	−0.012	0.022	−0.532	0.600	0.280	0.590
	Sex ^X^ Wind Speed	0.200	0.144	1.384	0.178	1.920	0.170
	**Best Fit Model (AIC = 107.53)**						
	Intercept	−1.143	0.622	−1.836	0.067		
	Temperature	0.091	0.023	3.978	<0.001	15.830	0.000
	Wind Speed	0.246	0.060	4.080	<0.001	16.650	0.000
